# Cerebral abscess complicating embolization of an arteriovenous malformation: Case report and review of litera

**Published:** 2014-07-04

**Authors:** Alireza Khoshnevisan, Askar Ghorbani, Narges Sistany Allahabadi, Farshid Farzaneh, Sina Abdollahzadeh, Sepehr Soleymani, Vahidreza Ostovan

**Affiliations:** 1 Department of Neurosurgery, Shariati Hospital, Tehran University of Medical Sciences, Tehran, Iran; 2 Department of Neurology, Shariati Hospital, Tehran University of Medical Sciences, Tehran, Iran; 3 Student of Medicine, School of Medicine, Tehran University of Medical Sciences, Tehran, Iran; 4 Department of Neurology, Shariati Hospital, Tehran University of Medical Sciences AND Iranian Center of Neurological Research, Tehran, Iran

**Keywords:** Brain Abscess, Arteriovenous Malformation, Endovascular Procedure

## Abstract

Central nervous system infection is a rare complication of endovascular procedures. We report a 21-year-old woman presented with headache, nausea, vomiting, and right-sided hemiparesis 4 months after endovascular embolization of cerebral arteriovenous malformation. Investigations led to the diagnosis of multiple brain abscesses. This is the sixth case report of brain abscess following endovascular interventions.

## Introduction

Endovascular intervention is effective and safe method for treating cerebral vascular disease; that being done frequently nowadays.^1^ Different kinds of materials such as N-butyl cyanoacrylate (NBCA), polyvinyl alcohol, onyx, and absolute alcohol can be used for embolization of brain arteriovenous malformations (AVMs).^2^ Central nervous system (CNS) infections are rare complications^1^ and herein, we report the sixth case, a postpartum patient who developed a delayed multiloculated brain abscess after undergoing endovascular embolization of AVMs.

## Case Report

**Figure 1:**
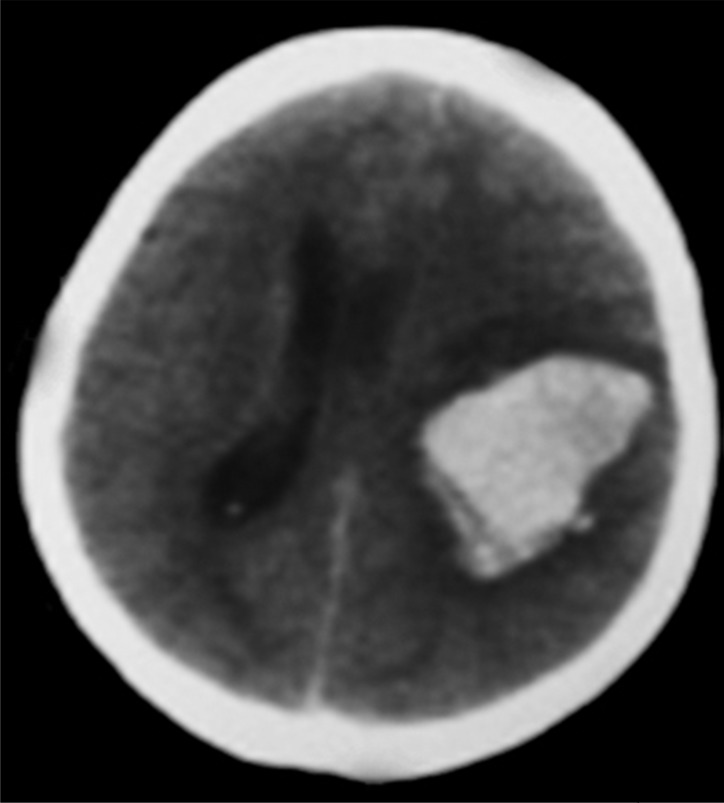
Brain computed tomography scan in the 4^th^ month of pregnancy showed left parietal lobe intra-cerebral hemorrhage

**Figure 2:**
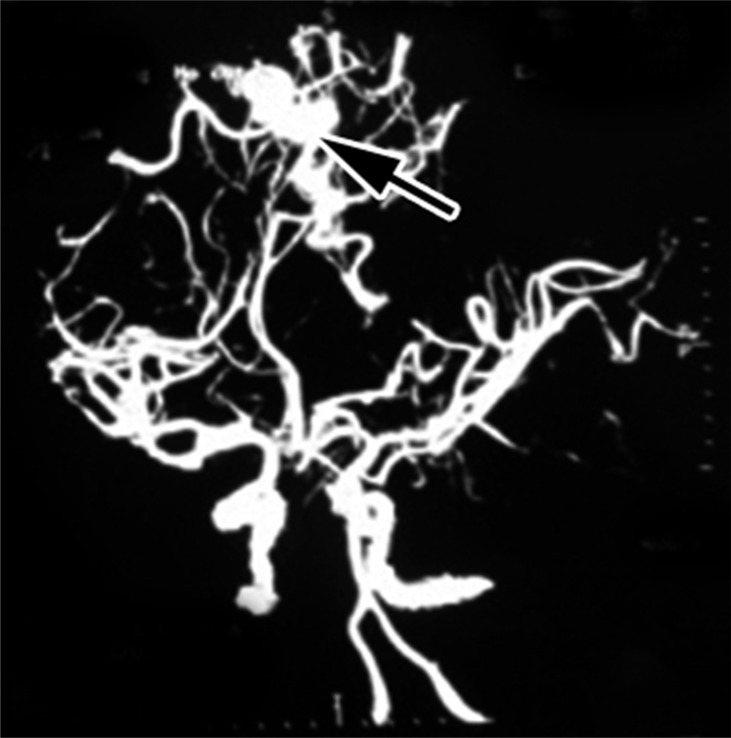
Magnetic resonance angiography revealed left frontoparietal arteriovenous malformation with feeders from anterior and middle cerebral arteries (arrow)

**Figure 3:**
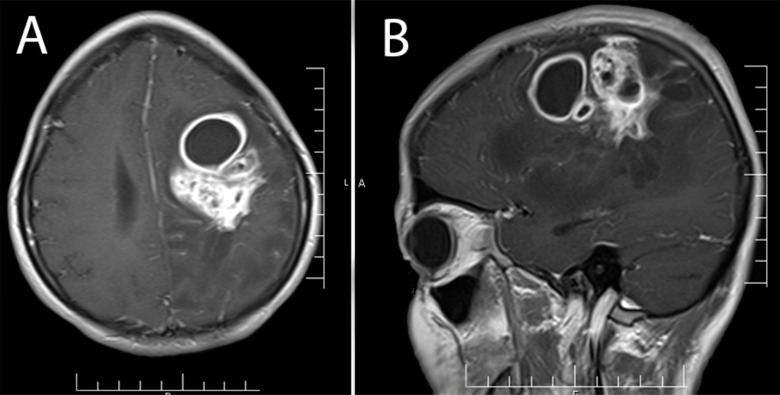
Axial (A) and sagittal (B) view of contrast-enhanced magnetic resonance imaging showed multiple ring enhancing lesions in the left frontoparietal

**Figure 4:**
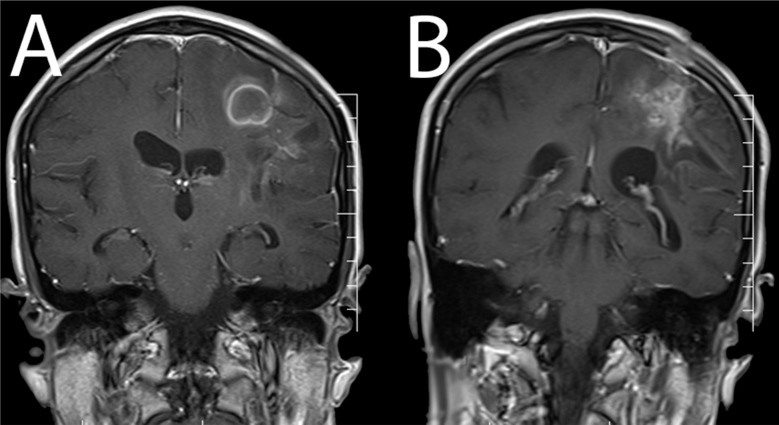
(A) Coronal view of contrast-enhanced magnetic resonance imaging one; (B) 4 months after drainage and antibiotic therapy of the abscesses

A 21-year-old woman presented with headache, nausea, vomiting, and right-sided hemiparesis 1 month after cesarean section. She had a history of thunderclap headache in the 4^th^ month of pregnancy, and further workup showed left parietal intra-cerebral hemorrhage (Figure 1); as a result of high flow AVM with feeders from left anterior and middle cerebral arteries (Figure 2). The AVM had been embolized completely using NBCA glue in the 6^th^ month of pregnancy. Furthermore, she had a history of urinary tract infection (UTI) in the 8^th^ month of pregnancy, and urine culture was positive for *Escherichia coli*. Neurological exam demonstrated right-sided facial palsy and hemiparesis, brisk deep tendon reflexes and upward plantar reflex on the right side. The rest of physical examination was unremarkable. Complete blood count showed mild leukocytosis (10,400/µl) with 81% polymorphonuclear cell. Brain magnetic resonance imaging (MRI) revealed multiple ring enhancing lesions in the left parietal with surrounding vasogenic edema in favor of brain abscess (Figure 3). Antibiotic therapy was started, and the largest showed *E. coli*. Intravenous antibiotic therapy was continued for 2 months. She improved over time, and the size of the lesions decreased in the follow-up MRI (Figure 4).

## Discussion

Although bacteremia has been shown to happen during therapeutic angiography at a rate of 32%, especially if lasting more than 2 h; only few cases of angiography induced brain abscess have been reported until now.^1^ Different mechanisms have been postulated to be responsible for brain abscess formation.^3^^-^^6^ Kelkar et al. retrospectively reviewed a total of 2918 cerebral angiograms and neurointerventional procedures which were done without prophylactic antibiotics, and showed that the risk of infection attributable to angiography was 0.1%.^7^ All infections were localized femoral artery infections with no systemic and central nervous system complications.^7^ They concluded that the overall risk of infection associated with most neuroangiographic procedures is very low. They also suggested that prophylactic antibiotics are probably not necessary for standard use in the situation of meticulous care during procedures, but they are better to be used for selected patients.^7^ Mourier et al.^3^ reported a 24-year-old patient with cerebral abscess several months after embolization of a right frontal AVM, similar to the present case. The recovered organism in that case was *Staphylococcus aureus*. They believed that the contamination of microcatheter or glue is responsible for the occurrence of infection. The infected and embolized AVM was removed due to poor response to antibiotic therapy, unlike to our presented case who had good response to antibiotic therapy and abscess drainage.^3^ A case of pyogenic cerebral abscess with superior sagittal sinus extension was reported by Pendarkar et al.^4^ He presented a 30-year-old male with right frontoparietal AVM who underwent multiple embolization and 6 months after the last embolization noticed a sinus with pus discharge over vertex. The causative pathogen was *Pseudomonas aeruginosa,* and they assumed that the bacteria had been carried to the site of infection through catheters or glue.^4^ Chagla and Balasubramaniam presented a case of brain abscess following AVM embolization and supposed that duration of the procedure, frequent handling of the catheters and the use of the large amount of foreign materials or NBCA may play an important role on the risk of infection during intervention.^5^ Similar to our reported cases, Sharma et al. presented a patient with headache and hemiparesis whom investigation showed brain abscess post-AVM embolization.^6^ He suggested that ischemic changes around the AVM after embolization may result in disruption of the blood brain barrier and subsequently brain abscess formation.^6^ In addition, he reported a case of brain abscess with *Burkholderia cepacia*, a low virulent organism and frequent colonizer of fluids (e.g., irrigation solutions); therefore, he concluded that contamination of irrigation fluids during embolization procedure and NBCA usage can lead to complications in the postoperative period.^6^ We present a patient with history of UTI and post-intervention brain abscess which the causative organisms were similar; as a result, we presumed that the presence of infectious foci in the other part of the body is another mechanism that can be responsible for cerebral abscess growth. This is the first case report of CNS infection following AVM embolization, which the same organism isolated from CNS and urogenital tract. We evacuated the abscess through a simple burr hole and drained the abscess by a brain cannula, unlike to some previously reported cases which necessitate the radical excision of the infected embolized AVM.^3^

## Conclusion

Development of CNS infection after endovascular procedure is an extremely rare event. Extreme care should be taken to ensure proper catheter handling and asepsis during embolization. Furthermore, investigation for active infection in other part of the body and treating properly before doing intervention is reasonable. 

## Notes

**How to cite this article: **Khoshnevisan A, Ghorbani A, Sistany Allahabadi N, Farzaneh F, Abdollahzadeh S, Soleymani S, et al. Cerebral abscess complicating embolization of an arteriovenous malformation: Case report and review of litera. Iran J Neurol 2014; 13(3): 181-4.
